# Effects of RNA structure and salt concentration on the affinity and kinetics of interactions between pentatricopeptide repeat proteins and their RNA ligands

**DOI:** 10.1371/journal.pone.0209713

**Published:** 2018-12-21

**Authors:** James J. McDermott, Bryce Civic, Alice Barkan

**Affiliations:** Institute of Molecular Biology, University of Oregon, Eugene, Oregon, United States of America; University of Surrey, UNITED KINGDOM

## Abstract

Pentatricopeptide repeat (PPR) proteins are helical repeat proteins that bind specific RNA sequences via modular 1-repeat:1-nucleotide interactions. Binding specificity is dictated, in part, by hydrogen bonds between the amino acids at two positions in each PPR motif and the Watson-Crick face of the aligned nucleobase. There is evidence that PPR-RNA interactions can compete with RNA-RNA interactions *in vivo*, and that this competition underlies some effects of PPR proteins on gene expression. Conversely, RNA secondary structure can inhibit the binding of a PPR protein to its specific binding site. The parameters that influence whether PPR-RNA or RNA-RNA interactions prevail are unknown. Understanding these parameters will be important for understanding the functions of natural PPR proteins and for the design of engineered PPR proteins for synthetic biology purposes. We addressed this question by analyzing the effects of RNA structures of varying stability and position on the binding of the model protein PPR10 to its *atpH* RNA ligand. Our results show that even very weak RNA structures (ΔG° ~ 0 kcal/mol) involving only one nucleotide at either end of the minimal binding site impede PPR10 binding. Analysis of binding kinetics using Surface Plasmon Resonance showed that RNA structures reduce PPR10’s on-rate and increase its off-rate. Complexes between the PPR proteins PPR10 and HCF152 and their respective RNA ligands have long half-lives (one hour or more), correlating with their functions as barriers to exonucleolytic RNA decay *in vivo*. The effects of salt concentration on PPR10-RNA binding kinetics showed that electrostatic interactions play an important role in establishing PPR10-RNA interactions but play a relatively small role in maintaining specific interactions once established.

## Introduction

Pentatricopeptide repeat (PPR) proteins comprise a large family of RNA binding proteins that function primarily in the context of mitochondrial and chloroplast gene expression [1, 2]. PPR proteins influence every RNA-mediated step in organellar gene expression, including RNA editing, group II intron splicing, RNA stability, and translation. Most PPR proteins act specifically on a handful of RNAs *in vivo*, and this functional specificity is reflected by sequence-specific RNA interactions *in vitro*. PPR proteins consist of tandem degenerate repeats of approximately 35-amino acids, each of which forms a helical hairpin. Consecutive repeats stack to form an elongated superhelix that binds single-stranded RNA. The sequence specificities of PPR proteins are, to some extent, predictable and customizable. Each repeat binds a single nucleotide, with nucleotide specificity dictated by the identities of two amino acids: the sixth amino acid in a given PPR motif and the first amino acid in the next (denoted as the 6 and 1’ amino acids according to the nomenclature in ref [3]). These two amino acids form a hydrogen bond network with the Watson-Crick face of the specified nucleotide [4, 5]. However, this “PPR code” is insufficient to fully explain the sequence specificities of natural PPR proteins, many of which have idiosyncratic features [6].

PPR proteins are found in all eukaryotes, but the family is particularly large in land plants, where it is made up of more than 400 members containing between two and approximately 30 PPR motifs. These can be divided into two subfamilies, termed P and PLS [7]. PLS proteins consist of variant repeat motifs and function primarily to specify sites of RNA editing [2]. P-type PPR proteins consist primarily of canonical “P-type” motifs, and are involved in group II intron splicing, transcript stabilization, and translational control. It is intriguing that proteins with this simple architecture can elicit such diverse effects on RNA. P-type PPR proteins have only rarely been observed to interact with other proteins [8]; instead, most functions of P-type PPR proteins may result from their capability to form an unusually long protein-RNA interface. For example, many PPR proteins with long repeat tracts stabilize RNA adjacent to their binding sites by blocking exoribonucleases (reviewed in [2]). Furthermore, sequestration of a long RNA segment by a PPR protein can influence local RNA folding [9], which, in turn, may influence RNA stability, processing, or translation.

PPR-RNA interactions are inhibited by RNA structures that involve nucleotides in the PPR binding site [6, 10, 11]. This inhibition is to be expected given that PPR motifs bind the Watson-Crick face of nucleobases. However, there is evidence that PPR proteins can bind *in vivo* to RNAs even when a portion of the binding site is complementary to an adjacent RNA sequence [9, 11]. For example, PPR53 binds the 5’-leader of the chloroplast pre-16S rRNA despite the fact that its binding site has the capability to form a hairpin (predicted ΔG° = -8 kcal/mol) with the adjacent RNA sequence [11]. In fact, the capability of PPR proteins to prevent the formation of RNA structures by occupying nucleotides within the potential structure is proposed to underlie the ability of PPR proteins to stimulate translation and group II intron splicing [9].

To develop a better understanding of the parameters that influence the ability of a PPR protein to compete with RNA for access to its binding site, we analyzed the effects of RNA structures of varying stabilities and position on a particularly well-characterized PPR-RNA interaction: that between the maize protein PPR10 and its *atpH* RNA ligand. Our experiments include the use of Surface Plasmon Resonance (SPR) to examine the kinetics of PPR:RNA interactions, a parameter that is likely to impact the biological functions of PPR proteins and that has, to our knowledge, not been reported previously.

## Results and discussion

### Impact of RNA secondary structure on PPR10 binding affinity

**Fig 1:**
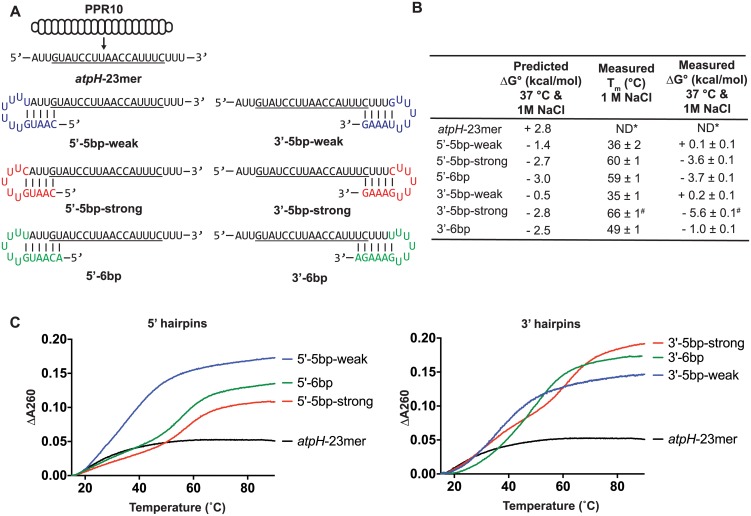
RNAs used to assess the effects of RNA secondary structure on PPR10 binding. (A) Sequences and predicted secondary structures of the RNA ligands. PPR10 is shown aligned to its 23-nt *in vivo* footprint near *atpH* (*atpH*-23mer). PPR10’s minimal binding site is underlined [9]. The *atpH*-23mer is not predicted to form any structure. Nucleotides that are appended to the PPR10 footprint to introduce RNA structure are colored. (B) Predicted and measured stabilities of each RNA structure at 1 M NaCl and 2.5 μM RNA. Predictions were made with mFold [16], which predicted only one structure for each RNA. The measured T_m_ and ΔG° values were calculated based on thermal melting curves (n = 3, +/- standard error of the mean). Values obtained at 180 mM NaCl, at different RNA concentrations, and from assays performed in reverse (transitions from high to low temperature) are shown in S1A Fig. *ND- Not determined due to lack of detectable structure. ^#^The measured values for the 3’-5bp-strong RNA are based on a single inflection point at 66°C, but the melting curve is biphasic (see panel C). Therefore, these values exaggerate the stability of this structure. (C) Representative melting curves at 1 M NaCl and 2.5 μM RNA.

We selected PPR10 to explore the interplay between RNA folding and PPR binding because PPR10’s functions, structure, and sequence specificity have been well characterized [4, 6, 9, 12–15]. PPR10 consists of 19 tandem PPR motifs flanked by capping helices. PPR10 localizes to chloroplasts, where it binds three sites that map in untranslated regions near the *atpH*, *psaJ*, and *psaI* genes. Of these, the site in the *atpH* 5’ UTR binds PPR10 with highest affinity, and these interactions have been most thoroughly characterized *in vitro*. PPR10’s minimal binding site at *atpH* spans 17-nucleotides, whereas its footprint (the region it protects from exoribonucleases) spans ~23 nucleotides (see Fig 1A). When PPR10 binds this site *in vivo*, it blocks exoribonucleases intruding from both the 5’- and 3’-directions and it also stimulates *atpH* translation. *In vitro* experiments provided evidence that PPR10 activates translation by sequestering RNA that would otherwise form an inhibitory secondary structure (ΔG° = -2.8 kcal/mol) with the *atpH* ribosome binding site [9].

To assess the influence of RNA secondary structure on PPR10-RNA interactions, we designed a series of RNAs harboring the PPR10 *atpH* footprint flanked by stem-loops whose stems include nucleotides at either end of the PPR10 binding site (Fig 1A). Constructs were designed such that they had only one predicted structure. Loops were composed solely of uridines to minimize interactions with other nucleotides in the RNA. We sought to distinguish how the number of binding-site nucleotides sequestered in the stem, the position of those nucleotides in the binding site, and the thermodynamic stability of the RNA structure impact PPR10 binding. Toward that end, we designed RNA hairpins of varying predicted thermodynamic stabilities that intrude on the PPR10 binding site to varying extents (Fig 1A and 1B). To distinguish effects of hairpin stability from effects of hairpin position, we designed both “strong” and “weak” RNA structures to sequester the same nucleotides at each end of the binding site.

We performed thermal denaturation experiments to validate the predicted thermodynamic stabilities of each RNA hairpin (Fig 1C and S1A Fig). Despite some discrepancies between the predicted and measured values, the data confirmed the intended trends in that the “strong” hairpins were considerably more stable than were the “weak” hairpins (Fig 1B). The discrepancy between the predicted and measured values was greatest for the 3’-5bp-strong RNA. However, the melting curve for this RNA had two inflection points, implying a complex folding pathway (Fig 1C). The measured ΔG° value for this RNA was calculated solely from the inflection point at the higher temperature and therefore exaggerates the stability of this structure. Similar T_m_ values were obtained from melting curves performed at other RNA concentrations, indicating that the melting profile was due to intramolecular structure and not intermolecular interactions (S1A Fig).

**Fig 2:**
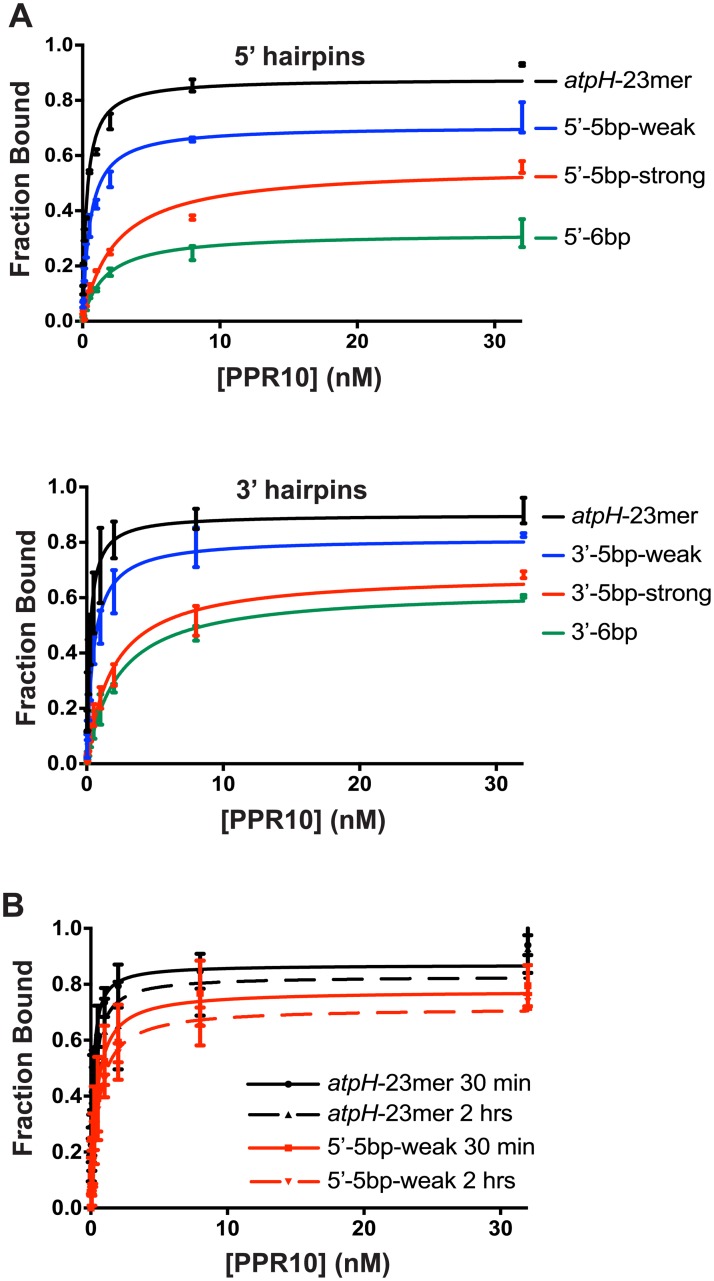
Gel mobility shift assays demonstrating effects of RNA structure on PPR10 binding. The RNAs (5 pM) are diagrammed in Fig 1A. PPR10 was used at concentrations of 32 nM, 8 nM, 2 nM, and six additional 2-fold dilutions. Data for replicate assays (n = 2) are shown as separate points connected by a vertical line. (A) Summary of binding data for reactions incubated for 30 minutes. Representative gels are shown in S1B Fig. (B) Comparison of results for binding reactions incubated for 30 minutes and 2 hours.

We then used gel mobility shift assays to examine the impact of each RNA structure on PPR10 binding affinity (Fig 2A). All of the structures inhibited PPR10 binding to some extent, with the degree of inhibition roughly correlating with the stability of the hairpin. The data show that even the 5’-5bp-weak and 3’-5bp weak structures (measured ΔG° ~ 0 at 1 M NaCl), which include just one nucleotide of the minimal binding site, had an impact on PPR10 binding. The predicted and measured stabilities of the 5’-5bp-strong and 5’-6bp hairpins were very similar (Fig 1B), yet the latter structure, which includes an additional nucleotide of PPR10’s minimal binding site, was substantially more inhibitory (Fig 2A). Thus, both the stability of the hairpin and its extent of intrusion on the minimal binding site impact the magnitude of inhibition. We had hoped to address whether binding is differentially affected depending on which end of the binding site is sequestered in the RNA stem. However, the measured free-energies were not closely matched for the 5’ and 3’constructs so we were unable to make firm conclusions in this regard.

The maximum fraction of RNA bound was reduced in rough proportion to the stability of the RNA hairpin (Fig 2A), consistent with the anticipated competition between intramolecular RNA interactions and RNA-protein interactions. To determine whether PPR10 can capture additional RNA over time as the RNA transiently unfolds, we determined whether the maximum amount of the 5’-5bp-weak RNA bound to PPR10 increased when the binding reaction was extended from 30 minutes to 2 hours (Fig 2B). The binding curves resulting from the two incubation times were very similar, indicating that the competing binding reactions (PPR10:RNA and intramolecular RNA:RNA) had reached equilibrium by 30 minutes.

### Kinetics of PPR-RNA interactions

**Fig 3:**
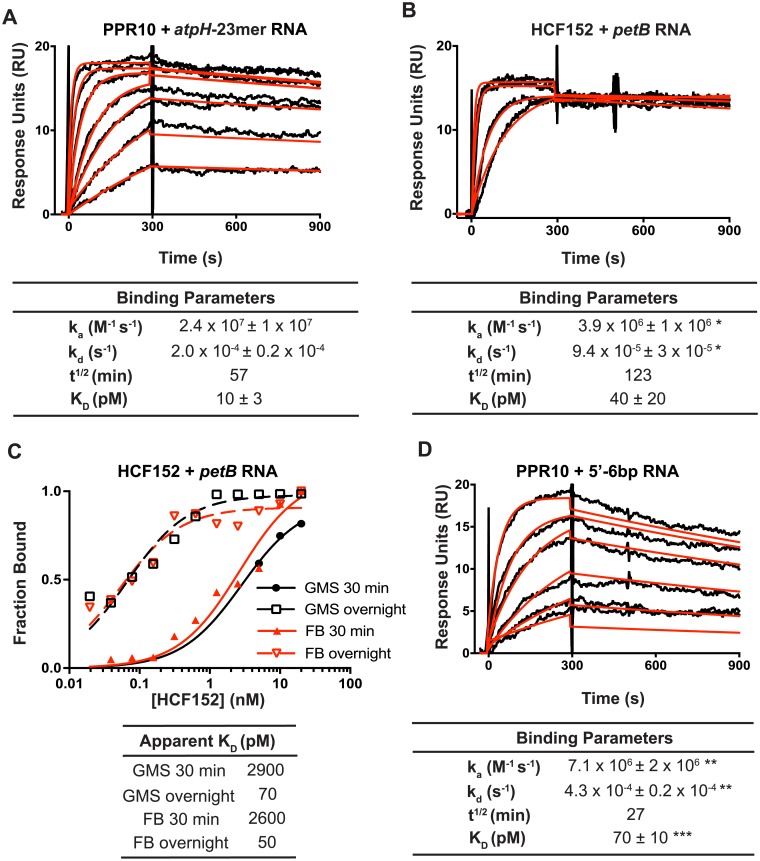
Analysis of PPR-RNA interactions by SPR. (A) SPR analysis of PPR10-*atpH* RNA interactions. Representative sensorgrams are shown at top. The data (black) were fit with a 1:1 Langmuir binding model (red). The RNAs are diagrammed in Fig 1A. PPR10 was used at a concentration of 5 nM and 2-fold dilutions thereof. Values in the table (+/- standard error of the mean) were calculated from data from three replicate experiments. A negative control demonstrating specificity of PPR10 for *atpH* RNA is shown in S2A Fig. Residuals are shown in S2C Fig. (B) SPR analysis of interactions between HCF152 and *petB* RNA. HCF152 was used at a concentration of 40 nM and 2-fold dilutions thereof. Representative sensorgrams are shown. Values in the table (+/- standard error of the mean) were calculated from four replicate experiments. A negative control demonstrating the specificity of HCF152 for *petB* RNA is shown in S2A Fig. Residuals are shown in S2C Fig. * Significantly different from data for the PPR10-atpH RNA interaction (P<0.05 according to a students t-test). (C) HCF152-*petB* RNA binding curves generated from gel mobility shift (GMS) and filter binding (FB) assays, comparing results from 30 min or overnight (~13 h) binding reactions. Examples of the raw data are shown in S2D Fig. (D) Effects of RNA structure on PPR10-RNA binding kinetics. The data are displayed as in panel (A). Values that are significantly different from those for the *atpH*-23mer are indicated (** = P<0.01, *** = P<0.001, according to a ratio paired t-test).

The kinetics of the interactions between PPR proteins and their RNA ligands are likely to impact the outcome of competing protein-RNA and RNA-RNA interactions, and the effects of those interactions on gene expression. To our knowledge, kinetic parameters for PPR-RNA interactions have not been reported. We hypothesized that the long binding interface expected for many PPR-RNA complexes would lead to slower off-rates in comparison with proteins that contact fewer nucleotides. We used SPR to determine on- and off- rates for two PPR-RNA complexes: (i) PPR10 and its *atpH* binding site, and (ii) HCF152 and its binding site in the chloroplast *psbH-petB* intercistronic region (Fig 3). HCF152 leaves a footprint of ~19-nucleotides and blocks 5’- and 3’-exoribonucleolytic degradation *in vivo* [17, 18], similar to PPR10’s effect near *atpH*. These proteins were chosen for the SPR experiments because they express well as recombinant proteins, their native binding sites are well defined, and they have been shown to bind with high affinity and specificity to those RNA sequences *in vitro* [9, 17].

The on- and off-rates for the PPR10-*atpH* RNA interaction predict an equilibrium constant (K_D_) of ~ 10 pM (Fig 3A), slightly lower than that inferred from equilibrium gel-mobility shift assays with the same protein preparation (S2E Fig, K_D_ ~50 pM). The inclusion of heparin in the gel-mobility shift binding assays may account for the higher apparent K_D_. Both the on-rate and off-rate for the HCF152-*petB* RNA interaction were several-fold slower than the those for the PPR10-*atpH* interaction (Fig 3B). These differences might reflect the different nucleotide compositions of the binding sites and/or idiosyncrasies of each protein’s PPR motifs. Notably, the K_D_ calculated from the measured kinetic parameters for HCF152-*petB* was roughly 30-fold lower than that we inferred from gel mobility shift assays [17]. Given the long half-life of the complex (approximately 2 hours), we wondered whether the prior assays had not reached equilibrium and had therefore underestimated binding affinity. To address this possibility, we compared the results of gel mobility shift and filter binding assays incubated for either 30 minutes (as in the prior study) or overnight (Fig 3C). The 30-minute incubation resulted in an apparent K_D_ similar to that reported previously, whereas the overnight incubation resulted in an apparent K_D_ similar to that inferred from kinetic data (Fig 3B). These results highlight the importance of ensuring that binding reactions have reached equilibrium when measuring K_D_’s for PPR-RNA interactions.

The kinetic parameters for the PPR10-*atpH* interaction are similar to those reported for the U1A RRM domain and its RNA ligand [19, 20]. However, the on- and off- rates for the HCF152-*petB* interaction were slower than those for PPR10 and U1A with their cognate RNAs. Taken together, these data suggest that some PPR tracts form unusually long-lived complexes with their RNA ligand, but there is not a simple relationship between the length of the RNA-protein interface and the life-time or affinity of the complex.

#### Effects of RNA structure on the kinetics of PPR10-RNA interactions

We next addressed the effects of RNA structures sequestering a portion of the PPR10 binding site on the kinetics of the PPR10-*atpH* RNA interaction. An RNA hairpin that includes two nucleotides of PPR10’s minimal binding site at the 5’-end (5’-6bp) decreased the on-rate and increased the off-rate several fold (Fig 3D). Analogous results were obtained with an RNA harboring a similar structure at the 3’-end (3’-6bp, see S2B Fig); however, this RNA was tethered to the sensor chip at the opposite end from the other RNAs we examined by SPR, and this may impact the binding kinetics. In any case, reduced on-rates are anticipated to result from the competition with adjacent RNA for PPR10 access to its binding site. The accelerated off-rates suggest that the PPR-nucleotide interactions at each end of the PPR-RNA complex can breathe, allowing the intramolecular RNA duplex to intrude on the PPR10 binding site. The fact that deletion of one or two nucleotides at either end of the minimal binding site prevents any apparent PPR10 interaction in gel mobility shift assays [9] argues against the possibility that substantive interactions can be established without the participation of the two nucleotides at either end of the binding site. Therefore, we favor an interpretation in which the RNA bound to PPR10 can exchange partners to form a competing intramolecular RNA interaction in a manner that is analogous to branch migration at the borders of alternative nucleic acid duplexes, and that once the RNA structure is established the protein dissociates and rarely rebinds in the context of the SPR assay.

### Contribution of electrostatic interactions to establishing and maintaining the PPR10-RNA complex

**Fig 4:**
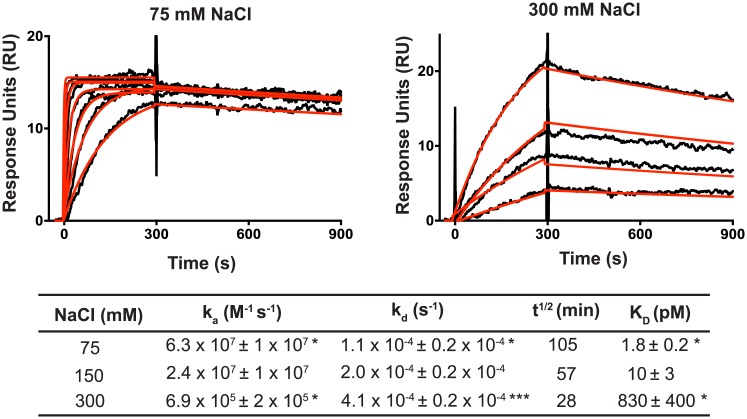
Effect of salt concentration on the kinetics of PPR10-atpH RNA interactions. Representative sensorgrams are shown at top. PPR10 was used at 5 nM and two-fold dilutions thereof. The data (black) were fit with a 1:1 Langmuir binding model (red). Residuals are shown in S2C Fig. The table below shows the binding parameters inferred from the data (average of three replicate experiments +/- standard error). Values that show a significant difference from those at 150 mM NaCl (see Fig 3A) are indicated (* = P<0.05, *** = P<0.001, according to a students t-test).

The k_a_ for the PPR10-*atpH* RNA interaction (~2 x 10^7^ M^-1^s^-1^, see Fig 3A) is considerably faster than that of diffusion-limited macromolecular interactions (~10^6^ M ^−1^ s^−1^) [21], suggesting that electrostatic interactions drive encounters between the protein and RNA. Indeed, the consensus PPR motif used in synthetic PPR designs includes a lysine residue that forms a salt-bridge to the phosphate backbone of bound RNA, and replacement of this lysine with alternative amino acids eliminates RNA binding [5, 22]. A basic “stripe” formed by lysines and arginines at this position is apparent also in the PPR10 crystal structure [4]; however, artifactual protein dimerization in the PPR10-RNA crystal structure [4, 13] prevents inferences from that structure about electrostatic contributions to PPR10:RNA binding. To explore how electrostatic forces influence the kinetics of PPR-RNA interactions, we used SPR to monitor the effect of varying salt concentration on the on- and off-rate of PPR10-*atpH* RNA interactions (Fig 4). An increase in salt concentration increases the electrostatic shielding around charged molecules, thereby decreasing their electrostatic interactions with other molecules. Increasing the NaCl concentration from 75 mM to 300 mM caused a dramatic increase in the K_D_ of the PPR10-RNA interaction. This was largely due to an effect on the on-rate, which decreased approximately 100-fold. By contrast, the off-rate increased only ~4-fold.

These results suggest that electrostatic forces make a large contribution to establishing interactions between PPR tracts and RNA, but make only a modest contribution to maintaining specific PPR-RNA interactions once established. These trends are similar to those obtained for several proteins with RRM domains [19, 20]. It is intriguing in this context that the “stripe” of positive surface potential adjacent to the RNA binding groove in consensus PPR tracts is flanked by a stripe of negative surface potential [5, 22]. Thus, the electrostatic steering that drives encounters between PPR tracts and RNA likely involves both attractive and repulsive forces that cooperate to optimize the alignment between the specificity determining amino acids and their cognate nucleobases.

## Summary

Results presented here provide insight into biophysical parameters that influence sequence-specific interactions between PPR proteins and RNA. Our results suggest that non-specific electrostatic interactions drive PPR proteins towards RNA, and that stable binding to specific sequences is established only when most of the binding site is single-stranded. Once established, the complexes between long PPR tracts (such as those in HCF152 and PPR10) and their cognate RNAs are long-lived, and this likely underlies their effectiveness as barriers to exoribonucleases. However, establishment of these interactions is inhibited by even very weak RNA secondary structures that intrude on the binding site. It is notable in this context that several PPR proteins have been shown to occupy RNA sites *in vivo* that are predicted to contribute to RNA hairpins that are substantially more stable than those analyzed here [9, 11, 23]. For example, the native PPR10 and PPR53 binding sites have the capacity to pair with adjacent RNA sequences to form structures whose stability is similar to or exceeds that of structures we found to be strongly inhibitory *in vitro* (ΔG° = -2.8 and -8.0 kcal/mol, respectively) [9, 11]. In fact, the binding sites of most PPR proteins are likely to have some degree of complementarity with nearby RNA sequences. Thus, it seems likely that RNA helicases and RNA chaperones facilitate PPR action *in vivo* by reducing secondary structures that would otherwise occlude their binding sites. Elucidating the nature of this interplay will be important for the design of synthetic PPR proteins and cognate binding sites, and offers an interesting area for future investigation.

## Materials and methods

### Protein expression and purification

PPR10 and HCF152 were expressed in *E*. *coli* as fusions with maltose binding protein (MBP), affinity purified on amylose resin, cleaved from the MBP moiety, and further purified by size exclusion chromatography as described previously [12, 17].

### RNA thermal melting assays

RNA thermal melting assays were performed as described in ref [24]. Free energies were inferred from the melting curves according to ref [25] using KaleidaGraph. RNAs were purchased from IDT. Assays performed in reverse (from high to low temperatures) and at varying RNA concentrations gave similar values (S1A Fig). Predicted free energies of RNA stem-loops were calculated from mFold (version 4.7) using the default parameters of 37 °C and 1M NaCl.

### Gel mobility shift assays

Gel mobility shift assays were performed as previously described [9], with minor modifications. In brief, synthetic RNA oligonucleotides (IDT) were 5’-end labeled with T4 polynucleotide kinase and [γ-^32^P]-ATP. The binding reactions contained 5 pM RNA, 40 mM Tris-HCl pH 7.5, 140 mM NaCl, 10% glycerol, 4 mM DTT, 10 U RNAsin, 0.1 mg/mL BSA, 0.5 mg/mL heparin, and protein at the indicated concentrations. Unless otherwise noted, binding reactions were incubated for 30 minutes at 25°C. Results were imaged with a Storm phosphorimager and quantified with Image Studio Lite. Curves were fit to the data using a nonlinear regression curve fit using Prism software. The sequences of the *atpH*-related RNAs are shown in Fig 1A. The *petB* RNA used in HCF152 binding assays had the following sequence: 5’ GGUAGUUCGACCGUGGA-3’.

### Surface Plasmon Resonance

Biotinylated RNAs with a standard 6-carbon linker were purchased from IDT, with the following sequences:

*atpH*: 5’-GAUUGUAUCCUUAACCAUUUCUUUU-3’ biotin;
3’-6bp: biotin5’-AUUGUAUCCUUAACCAUUUCUUUUUUUUUGAAAGA-3’
5’-6bp 5’-ACAAUGUUUUUUAUUGUAUCCUUAACCAUUUCUUU-3’-biotin;
*petB*: 5’-UGGUAGUUCGACGUGGAUUUU-3’-biotin.

SPR streptavidin chips (GE Sensor Chip SA) were labeled with 5 response units (RUs) of biotinylated RNA by injecting RNA (1 pM) in HBS buffer (100 mM HEPES pH 7.5, 150 mM NaCl, 3 mM EDTA, 1 mM ß-mercaptoethanol, 0.005% P20 surfactant) at a rate of 10 μL/s for 10 seconds. This yielded a maximum of approximately 25 Response Units (RUs) upon protein injection, the value suggested by Katsamba et al for analysis of high affinity interactions [26]. We used low RNA density on the chip in order to eliminate mass transport effects and ligand rebinding events during the dissociation phase [27, 28]. Prior to each experiment, the instrument was purged three times with fresh HBS buffer and equilibrated for several minutes to establish a flat baseline. For analyses of PPR10 interactions with RNAs harboring secondary structures, lane 1 was left blank for background subtraction, lane 2 was labeled with the *atpH* RNA, lane 3 was labeled with 5’-6bp RNA, and lane 4 was labeled with 3’-6bp RNA. Lanes 1–4 were analyzed in series with the same injections of PPR10 and the resulting data were statistically analyzed using a ratio paired t-test. For experiments that examined the effect of salt concentration on PPR10 binding kinetics, lane 1 was left blank for background subtraction and lane 2 was labeled with the *atpH* RNA; these data were statistically analyzed using a students t-test. For analyses of HCF152 with *petB*, lane 1 was left blank for background subtraction, lane 2 was labeled with *atpH* RNA and lane 4 was labeled with *petB* RNA; these data were statistically analyzed using a students t-test. The range of protein concentrations in each experiment were injected in a random order with buffer injections every third injection to be used as a second background subtraction. Bound proteins were washed off the chip between protein injections using 0.02% SDS in HBS buffer. Data were analyzed using the Biacore Evaluation software.

## Supporting information

S1 Fig (A) Predicted and measured stabilities of the *atpH*-related RNAs diagrammed in Fig 1A. (B) Representative gel mobility shift assays underlying the curves presented in Fig 2. See Fig 2 for details.
(PDF)

S2 Fig (A) Specificity controls for PPR10 and HCF152 SPR assays. Sensorgrams are shown for the analysis of PPR10 interaction with HCF152’s *petB* RNA ligand (left) and HCF152’s interaction with PPR10’s *atpH* RNA ligand (right). (B) SPR analysis of PPR10 interaction with the 3’-6bp RNA ligand (see Fig 1A). The experiment was performed as in Fig 3A except that the RNA was tethered to the SPR chip via biotin at its 5’-end. PPR10 was used at a concentration of 5 nM and 2-fold dilutions thereof. (C) Residuals for SPR assays. (D) Examples of gel mobility shift and filter binding data supporting the curves shown in Fig 3C. (E) Gel mobility shift assay of PPR10-*atpH* 23 mer interactions, using the same PPR10 protein preparation as used in the SPR assays. PPR10 was used at a concentration of 2.5 nM and 2-fold dilutions thereof.
(PDF)
