# Supplementary material for: Effects of RNA structure and salt concentration on the affinity and kinetics of interactions between pentatricopeptide repeat proteins and their RNA ligands
Source: PLoS One. 2018 Dec 21;13(12):e0209713. doi: 10.1371/journal.pone.0209713 (PMC6303017; doi:10.1371/journal.pone.0209713)
Supplement: S1 Fig — (A) Predicted and measured stabilities of the atpH-related RNAs diagrammed in Fig 1A. (B) Representative gel mobility shift assays underlying the curves presented in Fig 2. See Fig 2 for details. (PDF) [file pone.0209713.s001.pdf]

A

	T_m (°C) 1 M NaCl 2.5 μM RNA Reverse Melt	Measured ΔG (kcal/mol) 37 °C 1 M NaCl 2.5 μM RNA Reverse melt	T_m (°C) 1 M NaCl 5 μM RNA	Measured ΔG (kcal/mol) 37 °C 1 M NaCl 5 μM RNA	T_m (°C) 1 M NaCl 5 μM RNA Reverse Melt	Measured ΔG (kcal/mol) 37 °C 1 M NaCl 5 μM RNA Reverse melt	T_m (°C) 180 mM NaCl	Measured ΔG (kcal/mol) 25 °C 180 mM NaCl
atpH-23mer	ND*	ND*	ND*	ND*	ND*	ND*	ND*	ND*
5'-5bp-weak	37 ± 3	0.00 ± 0.1	37 ± 1	- 0.04 ± 0.06	35 ± 5	+ 0.04 ± 0.05	31 ± 3	- 0.4 ± 0.3
5'-5bp-strong	59 ± 1	- 3.5 ± 0.5	59 ± 1	- 3.5 ± 0.2	58 ± 1	- 3.5 ± 0.1	56 ± 1	- 4.2 ± 0.8
5'-6bp	57 ± 1	- 3.1 ± 0.1	58 ± 1	- 3.6 ± 0.2	57 ± 1	- 3.4 ± 0.2	52 ± 1	- 3.8 ± 0.1
3'-5bp-weak	33 ± 1	+ 0.3 ± 0.03	32 ± 1	+ 0.4 ± 0.1	33 ± 2	+ 0.3 ± 0.1	27 ± 7	+ 0.2 ± 0.5
3'-5bp-strong	64 ± 1	- 5.3 ± 0.1	67 ± 1	- 6.1 ± 0.2	64 ± 1	- 5.2 ± 0.1	63 ± 2	- 7.5 ± 1
3'-6bp	49 ± 1	- 1.0 ± 0.01	46 ± 1	- 1.0 ± 0.01	47 ± 1	- 1.0 ± 0.1	46 ± 1	- 1.7 ± 0.3

B

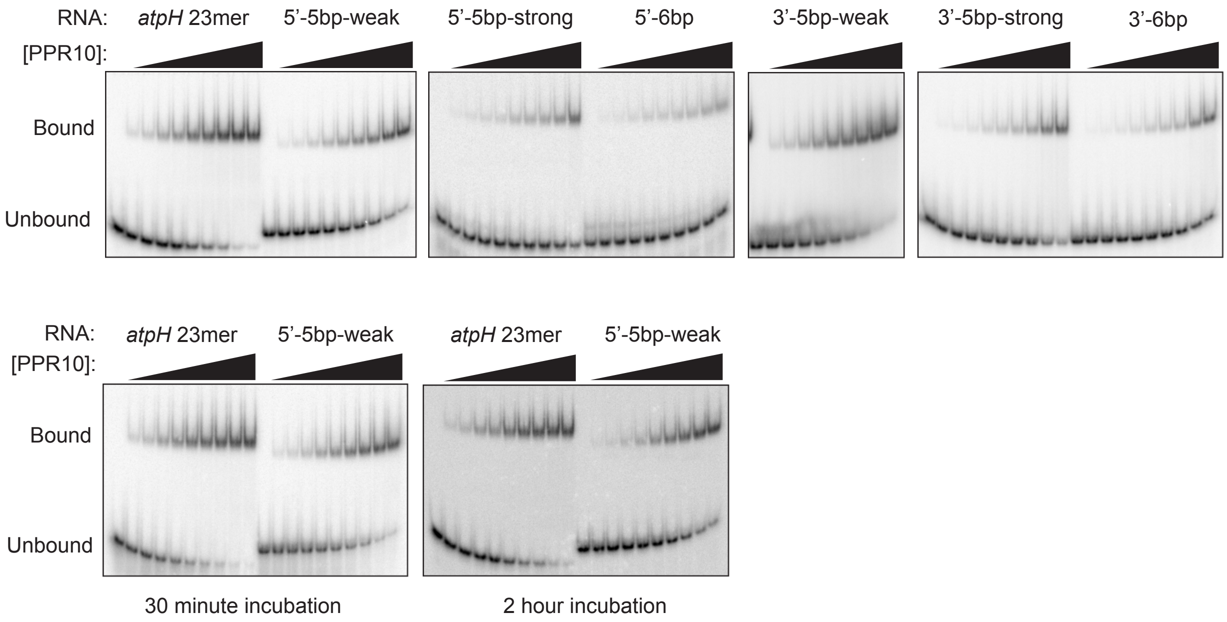

S1 Fig. (A) Predicted and measured stabilities of the atpH-related RNAs diagrammed in Fig 1A. (B) Representative gel mobility shift assays underlying the curves presented in Fig 2. See Fig 2 for details.
