# Supplementary material for: Effects of RNA structure and salt concentration on the affinity and kinetics of interactions between pentatricopeptide repeat proteins and their RNA ligands
Source: PLoS One. 2018 Dec 21;13(12):e0209713. doi: 10.1371/journal.pone.0209713 (PMC6303017; doi:10.1371/journal.pone.0209713)
Supplement: S2 Fig — (A) Specificity controls for PPR10 and HCF152 SPR assays. Sensorgrams are shown for the analysis of PPR10 interaction with HCF152’s petB RNA ligand (left) and HCF152’s interaction with PPR10’s atpH RNA ligand (right). (B) SPR analysis of PPR10 interaction with the 3’-6bp RNA ligand (see Fig 1A). The experiment was performed as in Fig 3A except that the RNA was tethered to the SPR chip via biotin at its 5’-end. PPR10 was used at a concentration of 5 nM and 2-fold dilutions thereof. (C) Residuals for SPR assays. (D) Examples of gel mobility shift and filter binding data supporting the curves shown in Fig 3C. (E) Gel mobility shift assay of PPR10-atpH 23 mer interactions, using the same PPR10 protein preparation as used in the SPR assays. PPR10 was used at a concentration of 2.5 nM and 2-fold dilutions thereof. (PDF) [file pone.0209713.s002.pdf]

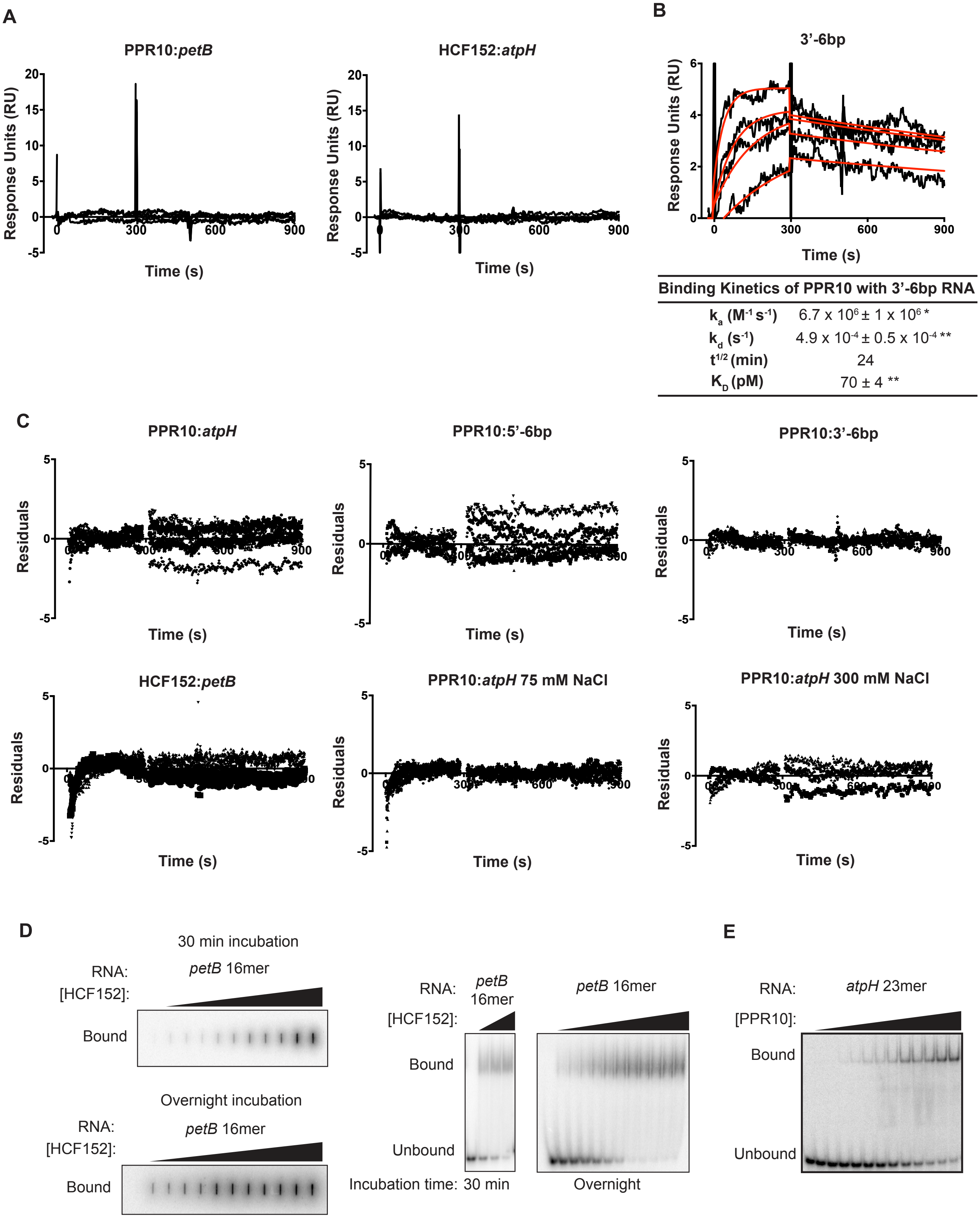

Binding Kinetics of PPR10 with 3’-6bp RNA	
k_a ($M^{-1}s^{-1}$)	$6.7 \times 10^6 \pm 1 \times 10^6$ *
k_d (s^{-1})	$4.9 \times 10^{-4} \pm 0.5 \times 10^{-4}$ **
$t^{1/2}$ (min)	24
K_D (pM)	70 ± 4 **

S2 Fig. (A) Specificity controls for PPR10 and HCF152 SPR assays. Sensorgrams are shown for the analysis of PPR10 interaction with HCF152’s petB RNA ligand (left) and HCF152’s interaction with PPR10’s atpH RNA ligand (right). (B) SPR analysis of PPR10 interaction with the 3’-6bp RNA ligand (see Fig 1A). The experiment was performed as in Fig 3A except that the RNA was tethered to the SPR chip via biotin at its 5’-end. PPR10 was used at a concentration of 5 nM and 2-fold dilutions thereof. (C) Residuals for SPR assays. (D) Examples of gel mobility shift and filter binding data supporting the curves shown in Fig 3C. (E) Gel mobility shift assay of PPR10-atpH 23 mer interactions, using the same PPR10 protein preparation as used in the SPR assays. PPR10 was used at a concentration of 2.5 nM and 2-fold dilutions thereof.
